# Supplementary material for: Disrupted network interactions serve as a neural marker of dyslexia
Source: Commun Biol. 2023 Nov 3;6:1114. doi: 10.1038/s42003-023-05499-2 (PMC10624919; doi:10.1038/s42003-023-05499-2)
Supplement: Supplementary file 2 — Supplementary Information [file 42003_2023_5499_MOESM2_ESM.pdf]

Supplementary Information

Turker et al. 2023 Communications Biology

Supplementary Table 1: Averages and standard deviations for in-scanner performance.

	Simple words	Complex words	Simple pseudowords	Complex pseudowords
Speech onset				
CG	0.796 (0.126)	0.853 (0.113)	0.927 (0.137)	1.149 (0.148)
DYS	0.915 (0.165)	1.037 (0.205)	1.125 (0.239)	1.258 (0.202)
Reading time				
CG	0.565 (0.097)	0.904 (0.144)	0.622 (0.101)	1.319 (0.208)
DYS	0.593 (0.097)	0.934 (0.121)	0.686 (0.097)	1.676 (0.265)
Accuracy				
CG	99.4 (1.2)	99.5 (1.1)	97 (2.7)	88.8 (7.9)
DYS	98.7 (2.8)	98.1 (2.4)	93 (7.5)	67.8 (18.4)

Speech onsets (in seconds), (overt) reading times (in seconds) and accuracy across all trials (percentage) for the control group (CG, n=27) and the dyslexia group (DYS, n=26).

Supplementary Table 2: Linear mixed model results for speech onsets (full sample comprising both groups: n=53).

Predictor	$\chi^2(1)$	t-value	p-value
Intercept	736.6	27.1	<0.001***
Complexity	43.2	6.6	<0.001***
Stimulus type	215.5	14.7	<0.001***
Group	8.5	2.9	0.0036**
Complexity: Stimulus type	173.9	13.2	<0.001***
Complexity: Group	24.5	5.0	<0.001***
Stimulus type: Group	38.3	6.2	<0.001***
Complexity: Stimulus type x Group	72.7	-8.5	<0.001***

Predictors, chi-squares, t- and p-values are provided for each effect and interaction (*p<0.05, **p<0.005, ***p<0.001).

Supplementary Table 3: Linear mixed model results for reading times (full sample comprising both groups: n=53).

Predictor	$\chi^2(1)$	t-value	p-value
Intercept	662.9	25.7	<0.001***
Complexity	1256.8	35.5	<0.001***
Stimulus type	35.5	6.0	<0.001***
Group	0.8	0.9	0.373
Complexity: Stimulus type	703.3	26.5	<0.001***
Complexity: Group	0.03	0.2	0.857
Stimulus type: Group	6.8	2.6	<0.001***
Complexity: Stimulus type x Group	229.2	15.1	<0.001***

Predictors, chi-squares, t- and p-values are provided for each effect and interaction (*p<0.05, **p<0.005, ***p<0.001).

Supplementary Table 4: **Linear mixed model results for reading accuracy (full sample comprising both groups: n=53).**

Predictor	$\chi^2(1)$	t-value	p-value
Intercept	8957.7	94.6	<0.001***
Complexity	<0.01	0.1	0.934
Stimulus type	7.0	-2.6	0.008**
Group	0.2	-0.5	0.631
Complexity: Stimulus type	42.8	-6.5	<0.001***
Complexity: Group	0.2	-0.5	0.631
Stimulus type: Group	6.9	-2.6	0.008**
Complexity: Stimulus type x Group	80.8	-8.9	<0.001***

Predictors, chi-squares, t- and p-values are provided for each effect and interaction (*p<0.05, **p<0.005, ***p<0.001).

Supplementary Table 5: Summary of stepdown results for group differences for speech onsets, reading times and accuracy (full sample comprising both groups: n=53).

	estimate	SE	z-ratio	sign. (p)
Speech onsets for simple words	-0.121	0.042	-2.908	0.004**
Speech onsets for complex words	-0.183	0.042	-4.406	<0.001***
Speech onsets for simple PW	-0.199	0.042	-4.787	<0.001***
Speech onsets for complex PW	-0.109	0.042	-2.630	0.008*
Reading times for simple words	-0.028	0.031	-0.891	0.373
Reading times for complex words	-0.030	0.031	-0.969	0.332
Reading times for simple PW	-0.063	0.031	-2.021	0.043*
Reading times for complex PW	-0.358	0.031	-11.409	<0.001***
Accuracy for simple words	0.007	0.015	0.480	0.6315
Accuracy for complex words	0.013	0.015	0.889	0.374
Accuracy for simple PW	0.041	0.015	2.724	0.006*
Accuracy for complex PW	0.210	0.015	13.979	<0.001***

Estimates, standard errors (SE), z-ratio and p-value are provided for each post-hoc comparison (*p<0.05, **p<0.005, ***p<0.001).

Supplementary Figure 1: Functional activation results for all four conditions (simple words, complex words, simple pseudowords, complex pseudowords) vs. rest in the control group.

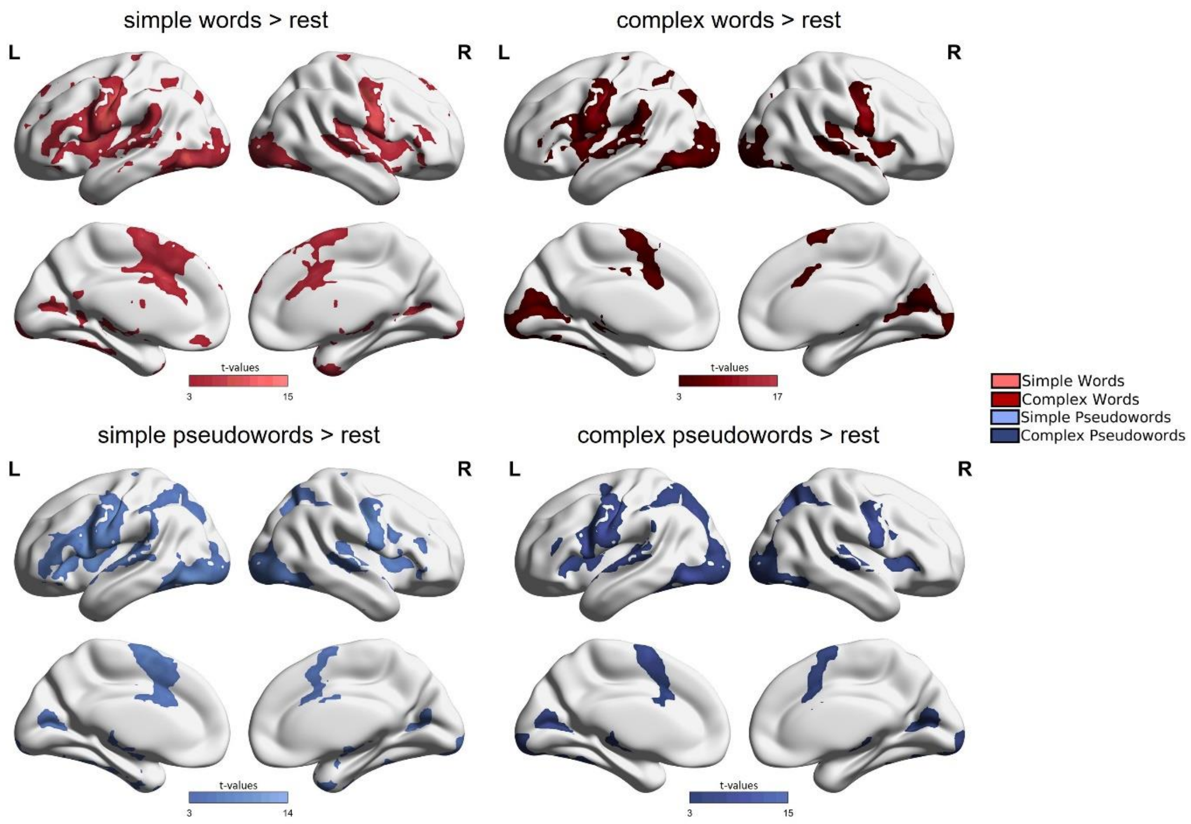

Colours illustrate activation during simple word (light red), complex word (dark red), simple pseudoword (light blue) and complex pseudoword reading (dark blue) ($p<0.001$ voxel-level, $p<0.05$ cluster-wise FWE corrected).

Supplementary Figure 2: Functional activation results for all four conditions (simple words, complex words, simple pseudowords, complex pseudowords) vs. rest in the dyslexia group.

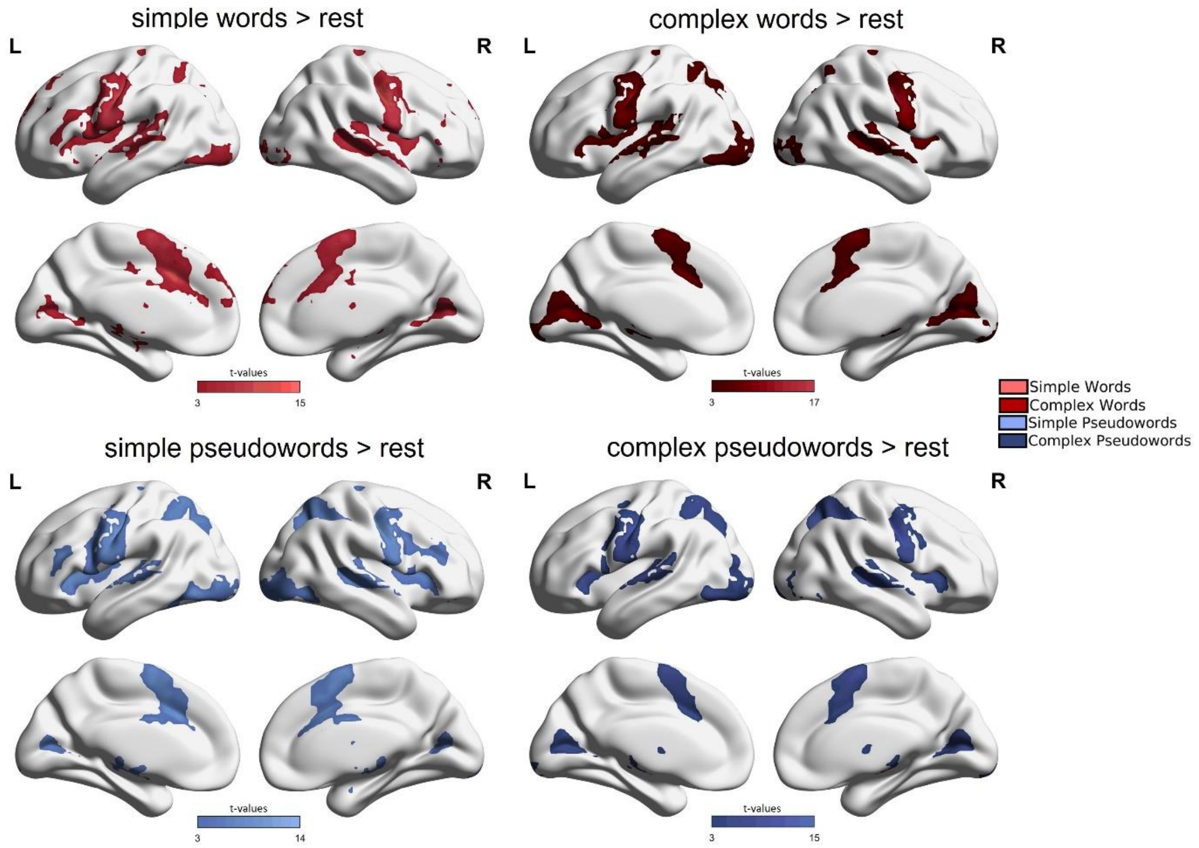

Colours illustrate activation during simple word (light red), complex word (dark red), simple pseudoword (light blue) and complex pseudoword reading (dark blue) ($p<0.001$ voxel-level, $p<0.05$ cluster-wise FWE corrected).

Supplementary Figure 3: Functional activation results for rest > all four conditions (simple words, complex words, simple pseudowords, complex pseudowords) in the control group.

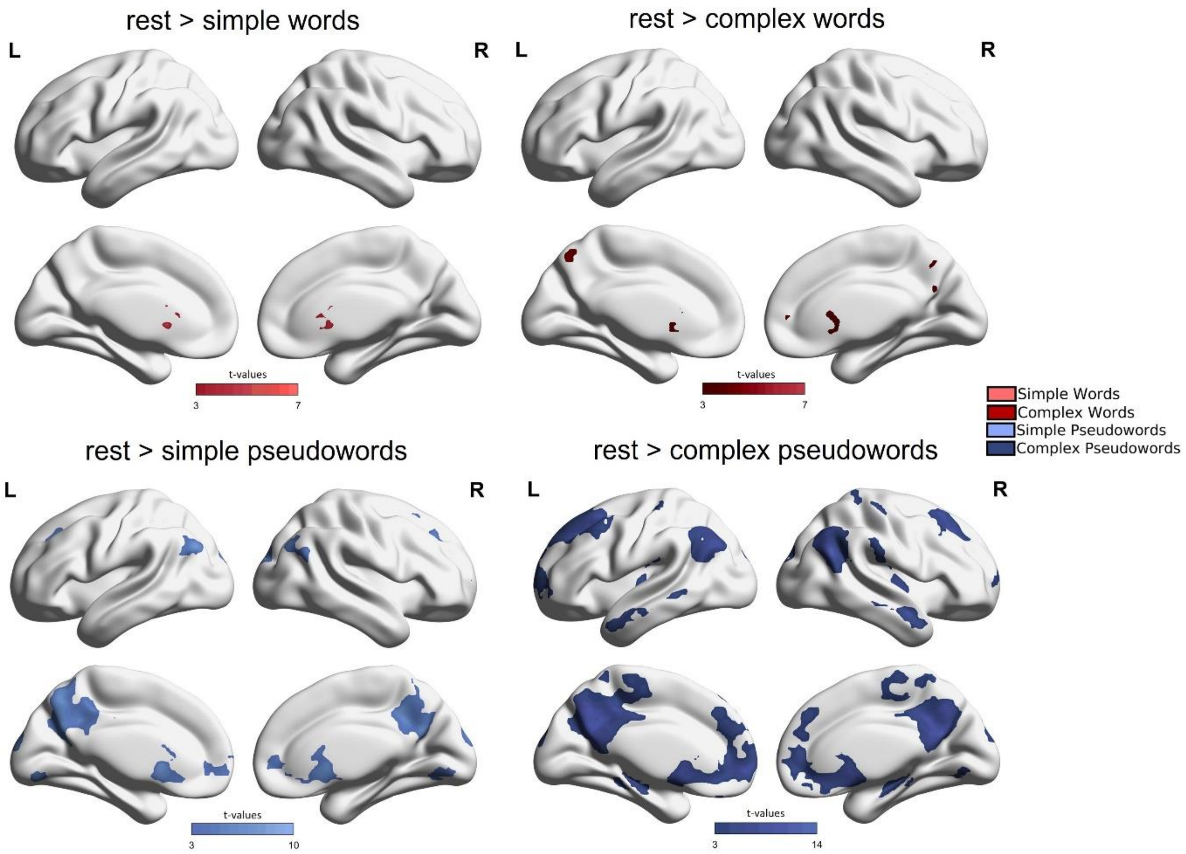

Colours illustrate activation during simple word (light red), complex word (dark red), simple pseudoword (light blue) and complex pseudoword reading (dark blue) ($p<0.001$ voxel-level, $p<0.05$ cluster-wise FWE corrected).

Supplementary Figure 4: Functional activation results for rest > all four conditions (simple words, complex words, simple pseudowords, complex pseudowords) in the dyslexia group.

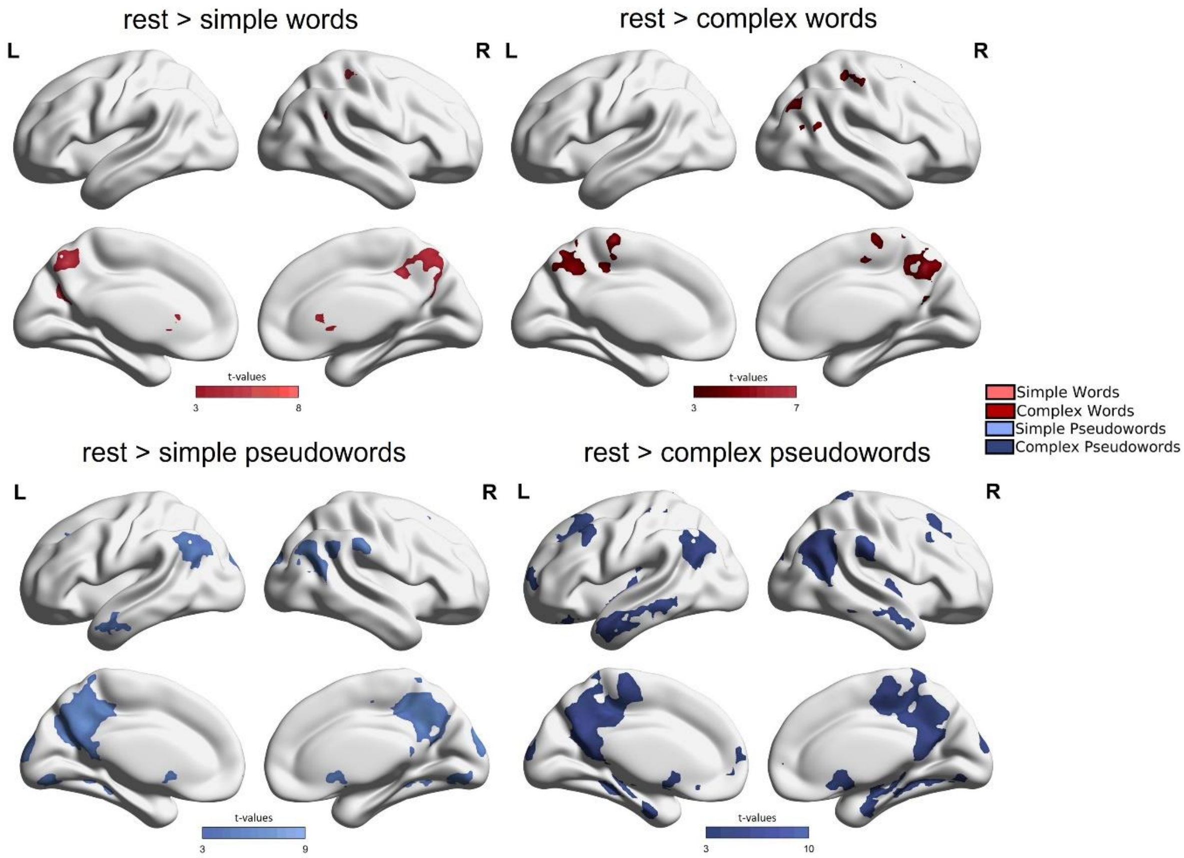

Colours illustrate activation during simple word (light red), complex word (dark red), simple pseudoword (light blue) and complex pseudoword reading (dark blue) ($p<0.001$ voxel-level, $p<0.05$ cluster-wise FWE corrected).

Supplementary Table 6: Comparison of brain activation between the control group (CG) and the dyslexia group (DYS) across contrasts.

Contrast	Cluster	k	HP	Brain area	MNI peak			t-value
					x	y	z	
CG > DYS all trials vs. rest	1	639	L	temporo-occipital/ lateral occipital/fusiform cortex	-54	-60	-4	5.66
	2	392	L	SMG/ SPL	-49	-43	51	5.23
	3	116	R	temporo-occipital/ lateral occipital/fusiform cortex	50	-50	-26	4.9
	4	93	R	cerebellum VIIIa/IIb/IX	18	-63	-48	5.02
	5	84	L/R	occipital fusiform/ lingual gyri	25	-85	-12	4.68
CG > DYS PW vs. rest	1	576	L	temporo-occipital/ lateral occipital/fusiform cortex	-57	-65	-2	5.82
	2	386	L	SMG	-49	-43	51	5.53
	3	170	R	cerebellum VIIIa/b/IX/II	20	-63	-46	5.00
	4	128	L/R	occipital fusiform/ occipital pole/ lingual gyri	25	-85	-12	4.85
	5	108	R	occipital fusiform cortex/ ITG cerebellum I	48	-53	-26	5.28
CG > DYS W vs. rest	1	497	L	temporo-occipital/ lateral occipital/fusiform cortex	-54	-60	-4	5.29
	2	204	L	SMG/SPL	-44	-50	62	4.93
CG > DYS PW vs. W	1	85	R	cerebellum VIIIa/b	28	-70	-51	4.67
	2	74	L	inferior lateral occipital/ temporo-occipital	-57	-65	-2	4.98

Only significant results are reported (voxelwise p<0.001, clusterwise FWE correction p<0.05). CG= control group, DYS= adults with dyslexia, HP= hemisphere, ITG = inferior temporal gyrus; k: cluster extent, L = left, MNI = Montreal Neurological Institute, R = right, W= word reading, PW = pseudoword reading.

Supplementary Table 7: Differences in functional connectivity from the core underactivated regions to other brain areas as revealed by PPI.

PPI seed	CG > DYS	k	HP	Brain area	MNI peak			t-value
					x	y	z	
L SMG	PW > rest	808	L/R	occipital pole/ superior lateral occipital	-9	-95	15	5.32
	W > rest	203	L	inferior lateral occipital/ temporo-occipital	-47	-60	-4	5.35
		124	R	cerebellum VIIIa/b/II	25	-65	-48	5.46
		69	L	cerebellum VIIIb/I/II	-22	-68	-46	4.30
L vOTC	PW > rest	370	L/R	cuneus/ superior lateral occipital	-4	-83	20	5.19
		317	R	angular gyrus / superior lateral occipital	50	-50	34	6.17
		166	R	occipital fusiform cortex	33	-80	-15	5.01
		108	L/R	paracingulate/ cingulate gyrus	-2	52	18	5.26
		82	R	pMTG	53	-13	-10	5.03
		68	L/R	superior frontal gyrus	6	24	64	5.27
		65	R	frontal pole	35	57	12	5.21
	W > rest	59	L	pSMG	-54	-45	34	4.44
	PW > W*	60	L/R	cuneus	3	-83	18	4.17
	*exact same cluster found in DYS > CG: W > PW							
R vOTC	PW > rest	113	L	Pre-/postcentral gyrus	-54	-11	40	4.38
		89	L	Inferior lateral occipital/ fusiform cortex	-42	-70	-12	5.21
		76	R	Inferior lateral occipital/ fusiform cortex	43	-75	-12	5.18
	W > rest	65	L	Precuneus	-17	-65	34	4.88
		57	R	SMA	6	-8	64	4.32
	DYS > CG W > PW	50	R	Cuneus/ Supracalcarine cortex	6	-83	20	4.32
R cerebellum	PW > rest	5425	L/R	Superior frontal/ postcentral/ SPL to vOTC	-19	-73	64	6.36
		518	L	Middle frontal/precentral gyrus	-44	2	56	5. 49
		355	L	IPL/pMTG	-67	-48	12	4.99
		214	L/R	Precuneus/cingulate gyrus	1	-43	-2	5.05
		177	R	Superior lateral occipital	30	-65	64	4.93
		126	R	STG	63	2	1	4.89
		120	R	Thalamus	8	-28	12	4.5
		118	L	Frontal pole	-49	49	-10	5.2
		90	R	Postcentral gyrus/ aSMG	60	-18	40	5.53
		65	L	IFG (Pars triangularis)	-49	24	1	4.46
		64	L	Frontal pole	-2	67	1	4.56
		60	R	OTC/fusiform cortex	40	-53	-24	4.35

W > rest	1490	L/R	SMA/ post-precentral gyri	-14	-6	40	5.11
	607	L	Precentral gyrus	-49	4	34	5.22
	154	L	Operculum/ STG	-64	-3	1	4.22
	100	R	MFG/ precentral gyrus	43	9	56	5.49
	77	L	OTC	-52	-53	-10	4.03
	77	R	Parietal operculum	35	-23	20	5.09
	73	L	Temporal pole/ frontal pole	-54	17	-10	4.72
	68	L	pSMG/ SPL	-44	-48	64	4.69
	59	L	aSMG	-67	-43	40	4.83
	59	R	Precentral gyrus	38	-16	45	5.51

Only significant results are reported (voxelwise $p<0.001$, clusterwise FWE correction $p<0.05$). Abbreviations: aSMG = anterior supramarginal, IFG = inferior frontal gyrus, IPL = inferior parietal lobe, MFG = middle frontal gyrus, pMTG = posterior middle temporal gyrus, pSMG = posterior supramarginal gyrus, SMA = supplementary motor area, SPL = superior parietal lobe, STG = superior temporal gyrus, OTC = occipito-temporal cortex,

Supplementary Table 8: Values for the DCM comprising left TPC, left IFG and left vOTC for the control group.

Connection	Intrinsic connectivity	Pp	Words	Pp	Pseudowords	Pp
L TPC → L IFG	**0.357 (0.001)**	**1**	**-0.498 (0.025)**	**0.981**	0 (0)	0
L TPC → L vOTC	**0.270 (0.001)**	**1**	**-0.43 (0.016)**	**0.989**	0 (0)	0
L IFG → L TPC	0 (0)	0	0 (0)	0.000	0.169 (0.041)	0.53
L IFG → L vOTC	-0.087 (0.002)	0.92	**0.567 (0.017)**	**0.998**	**0.511 (0.014)**	**0.99**
L vOTC → L TPC	**-0.182 (0.001)**	**1**	0 (0)	0.000	**0.398 (0.013)**	**0.99**
L vOTC → L IFG	**-0.224 (0.001)**	**1**	0 (0)	0.000	0 (0)	0

Intrinsic connectivity for each connection and the strength of the modulation (in Hz) during word or pseudoword reading are given with posterior probabilities (Pp). Significant effects (Pp>95) marked in bold.

Supplementary Table 9: Values for the DCM comprising left TPC, left IFG and left vOTC for the dyslexia group.

Connection	Intrinsic connectivity	Pp	Words	Pp	Pseudowords	Pp
L TPC → L IFG	**0.106 (0)**	**1**	0 (0)	0	**0.33 (0.016)**	**0.96**
L TPC → L vOTC	**0.135 (0.001)**	**1**	**-0.411 (0.018)**	**0.98**	0 (0)	0
L IFG → L TPC	**-0.128 (0.001)**	**1**	**0.827 (0.021)**	**1**	**0.729 (0.021)**	**1**
L IFG → L vOTC	**-0.198 (0.001)**	**1**	**0.456 (0.019)**	**0.99**	0.302 (0.021)	0.90
L vOTC → L TPC	**-0.125 (0.001)**	**0.99**	**0.656 (0.011)**	**1**	**0.513 (0.013)**	**1**
L vOTC → L IFG	**-0.106 (0.001)**	**0.99**	0 (0)	0	0.24 (0.018)	0.86

Intrinsic connectivity for each connection and the strength of the modulation (in Hz) during word or pseudoword reading are given with posterior probabilities (Pp). Significant effects (Pp>95) marked in bold.

Supplementary Table 10: Values for the group comparison DCMs (CG vs. DYS) for the classical reading network.

Connection	Intrinsic connectivity (group diff.)	Pp	Words (group diff.)	Pp	Pseudowords (group diff.)	Pp
L TPC → L IFG	**-0.118 (0)**	**1**	0 (0)	0	0 (0)	0
L TPC → L vOTC	-0.04 (0.001)	0.645	0 (0)	0	0 (0)	0
L IFG → L TPC	0 (0)	0	0.361 (0.028)	0.9	0 (0)	0
L IFG → L vOTC	0 (0)	0	0 (0)	0	0 (0)	0
L vOTC → L TPC	0 (0)	0	**0.431 (0.012)**	**1**	0 (0)	0
L vOTC → L IFG	0 (0)	0	0 (0)	0	0 (0)	0

Intrinsic connectivity for each connection and group differences are provided. Positive values in group differences (either for the intrinsic connectivity or the word/pseudoword modulation) indicate stronger effects in the dyslexia group (DYS > CG), negative values stronger effects in the control group (CG > DYS). We provide posterior probabilities (Pp). Significant effects (Pp>95) marked in bold.

Supplementary Table 11: Values for the DCM comprising left SMG, left vOTC, right vOTC and right cerebellum for the control group.

Connection	Intrinsic connectivity	Pp	Words	Pp	Pseudowords	Pp
L vOTC → L SMG	**0.151 (0.001)**	**1**	0 (0)	0	0 (0)	0
L vOTC → R vOTC	**0.097 (0.001)**	**0.97**	0 (0)	0	0 (0)	0
L vOTC → R Cer	-0.017 (0)	0.49	0 (0)	0	0 (0)	0
L SMG → L vOTC	0 (0)	0	0 (0)	0	0 (0)	0
L SMG → R vOTC	**0.097 (0.001)**	**0.98**	0 (0)	0	**0.356 (0.009)**	**1**
L SMG → R Cer	0 (0)	0	0 (0)	0	0 (0)	0
R vOTC → L vOTC	0.075 (0.002)	0.85	0 (0)	0	0.288 (0.013)	0.93
R vOTC → L SMG	0 (0)	0	0 (0)	0	0.161 (0.019)	0.67
R vOTC → R Cer	**0.08 (0)**	**1**	0.143 (0.017)	0.67	0 (0)	0
R Cer →L vOTC	**-0.23 (0.001)**	**1**	0 (0)	0	0.268 (0.035)	0.77
R Cer → L SMG	**-0.191 (0.001)**	**1**	0 (0)	0	0 (0)	0
R Cer → R vOTC	**-0.284 (0.001)**	**1**	**0.595 (0.018)**	**1**	0 (0)	0

Intrinsic connectivity for each connection and the strength of the modulation (in Hz) during word or pseudoword reading are given with posterior probabilities (Pp). Significant effects (Pp>95) marked in bold.

Supplementary Table 12: Values for the DCM comprising left SMG, left vOTC, right vOTC and right cerebellum for the dyslexia group.

Connection	Intrinsic connectivity	Pp	Words	Pp	Pseudowords	Pp
L vOTC → L SMG	**0.102 (0)**	**1**	0 (0)	0	**0.531 (0.01)**	**1**
L vOTC → R VOTC	0 (0)	0	**0.383 (0.012)**	**0.992**	**0.498 (0.013)**	**1**
L vOTC → R Cer	0.043 (0.001)	0.83	**0.432 (0.009)**	**1**	**0.487 (0.011)**	**1**
L SMG → L vOTC	-0.034 (0.001)	0.70	0 (0)	0	0 (0)	0
L SMG → R vOTC	0 (0)	0	0 (0)	0	0 (0)	0
L SMG →R Cer	0 (0)	0	0 (0)	0	-0.111(0.014)	0.55
R vOTC → L vOTC	0 (0)	0	**0.467 (0.013)**	**1**	**0.713 (0.015)**	**1**
R VOTC → L SMG	0 (0)	0	**-0.359 (0.01)**	**1**	**-0.394 (0.016)**	**0.99**
R vOTC → R Cer	0 (0)	0	0 (0)	0	0 (0)	0
R Cer → L vOTC	**-0.243 (0.001)**	**1**	0 (0)	0	0.392 (0.023)	0.947
R Cer → L SMG	**-0.092 (0)**	**1**	0 (0)	0	0 (0)	0
R Cer → R vOTC	**-0.148 (0.001)**	**1**	0.379 (0.032)	0.893	**0.651 (0.025)**	**1**

Intrinsic connectivity for each connection and the strength of the modulation (in Hz) during word or pseudoword reading are given with posterior probabilities (Pp). Significant effects (Pp>95) marked in bold.

Supplementary Table 13: Values for the group comparison DCMs (CG vs. DYS) for the DCM comprising left SMG, left vOTC, right vOTC and right cerebellum.

Connection	Intrinsic connectivity (group diff.)	Pp	Words (group diff.)	Pp	Pseudowords (group diff.)	Pp
L vOTC → L SMG	0 (0)	0	0 (0)	0	**0.267 (0.009)**	**1**
L vOTC → R vOTC	**-0.076 (0.001)**	**1**	0 (0)	0	0 (0)	0
L vOTC → R Cer	**0.042 (0)**	**1**	**0.262 (0.005)**	**1**	**0.201 (0.004)**	**1**
L SMG → L vOTC	-0.024 (0.001)	0.512	0 (0)	0	0 (0)	0
L SMG → R vOTC	0 (0)	0	0 (0)	0	0 (0)	0
L SMG → R Cer	0 (0)	0	0 (0)	0	0 (0)	0
R vOTC → L vOTC	-0.036 (0.001)	0.615	0 (0)	0	0.104 (0.014)	0.536
R vOTC → L SMG	0.024 (0.001)	0.582	0 (0)	0	**-0.272 (0.008)**	**1**
R vOTC → R Cer	**-0.063 (0)**	**1**	**-0.191 (0.005)**	**1**	0 (0)	0
R Cer → L vOTC	0 (0)	0	0 (0)	0	0 (0)	0
R Cer → L SMG	0 (0)	0	0 (0)	0	0 (0)	0
R Cer → R vOTC	0.024 (0.001)	0.512	0 (0)	0	0.157 (0.031)	0.544

Intrinsic connectivity for each connection and group differences are provided. Positive values in group differences (either for the intrinsic connectivity or the word/pseudoword modulation) indicate stronger effects in the dyslexia group (DYS > CG), negative values stronger effects in the control group (CG > DYS). We provide posterior probabilities (Pp). Significant effects (Pp>95) marked in bold.

Supplementary Table 14: Full table of Bayesian correlations for functional activation and reading performance (n=53).

ROI	Performance	τ	BF_{10}
L vOTC	pseudoword reading (SLRT)	0.323	52.775
	pseudoword speech onsets (MRI)	-0.133	0.463
	pseudoword reading times (MRI)	-0.273	9.748
	pseudoword accuracy (MRI)	0.304	25.109
	word reading (SLRT)	0.395	911.539
	word speech onsets (MRI)	-0.208	1.834
	word reading times(MRI)	0.015	0.182
	word accuracy (MRI)	0.242	4.135
L SMG	pseudoword reading (SLRT)	0.497	48686.612
	pseudoword speech onsets (MRI)	-0.309	30.107
	pseudoword reading times (MRI)	-0.422	2509.199
	pseudoword accuracy (MRI)	0.334	71.321
	word reading (SLRT)	0.404	1334.189
	word speech onsets (MRI)	-0.27	8.93
	word reading times(MRI)	0.017	0.183
	word accuracy (MRI)	0.148	0.584
R vOTC	pseudoword reading (SLRT)	0.309	33.531
	pseudoword speech onsets (MRI)	-0.094	0.288
	pseudoword reading times(MRI)	-0.252	5.386
	pseudoword accuracy (MRI)	0.272	9.341
	word reading (SLRT)	0.344	116.502
	word speech onsets (MRI)	-0.131	0.453
	word reading times(MRI)	0.008	0.181
	word accuracy (MRI)	0.173	0.887
R cerebellum	pseudoword reading (SLRT)	0.242	4.427
	pseudoword speech onsets (MRI)	-0.161	0.727
	pseudoword reading times (MRI)	-0.253	5.611
	pseudoword accuracy (MRI)	0.304	25.109
	word reading (SLRT)	0.276	11.466
	word speech onsets (MRI)	-0.21	1.897
	word reading times(MRI)	-0.08	0.254
	word accuracy (MRI)	0.083	0.259
L/R lingual	pseudoword reading (SLRT)	0.302	26.275
	pseudoword speech onsets (MRI)	-0.154	0.64
	pseudoword reading times (MRI)	-0.176	0.955
	pseudoword accuracy (MRI)	0.189	1.219
	word reading (SLRT)	0.347	364.659
	word speech onsets (MRI)	-0.262	7.203
	word reading times(MRI)	0.063	0.223
	word accuracy (MRI)	0.165	0.774

We correlated activation within the five hypoactive regions (left vOTC, left SMG, right vOTC, right cerebellum, and bilateral lingual gyri) with in- and out-of-scanner performance. Out-of-scanner performance comprised word and pseudoword reading as assessed by the SLRT-II, whereas in-scanner performance included pseudoword / word speech onsets, reading times and accuracy. Significant results with $BF_{10} > 7$ are provided in Table 2 in the Manuscript. Missing values: exclude cases pairwise.

Supplementary Figure 5: Correlational plots for functional activation in left vOTC for the respective contrasts (pseudowords > rest, words > rest) and behavioural performance (n=53).

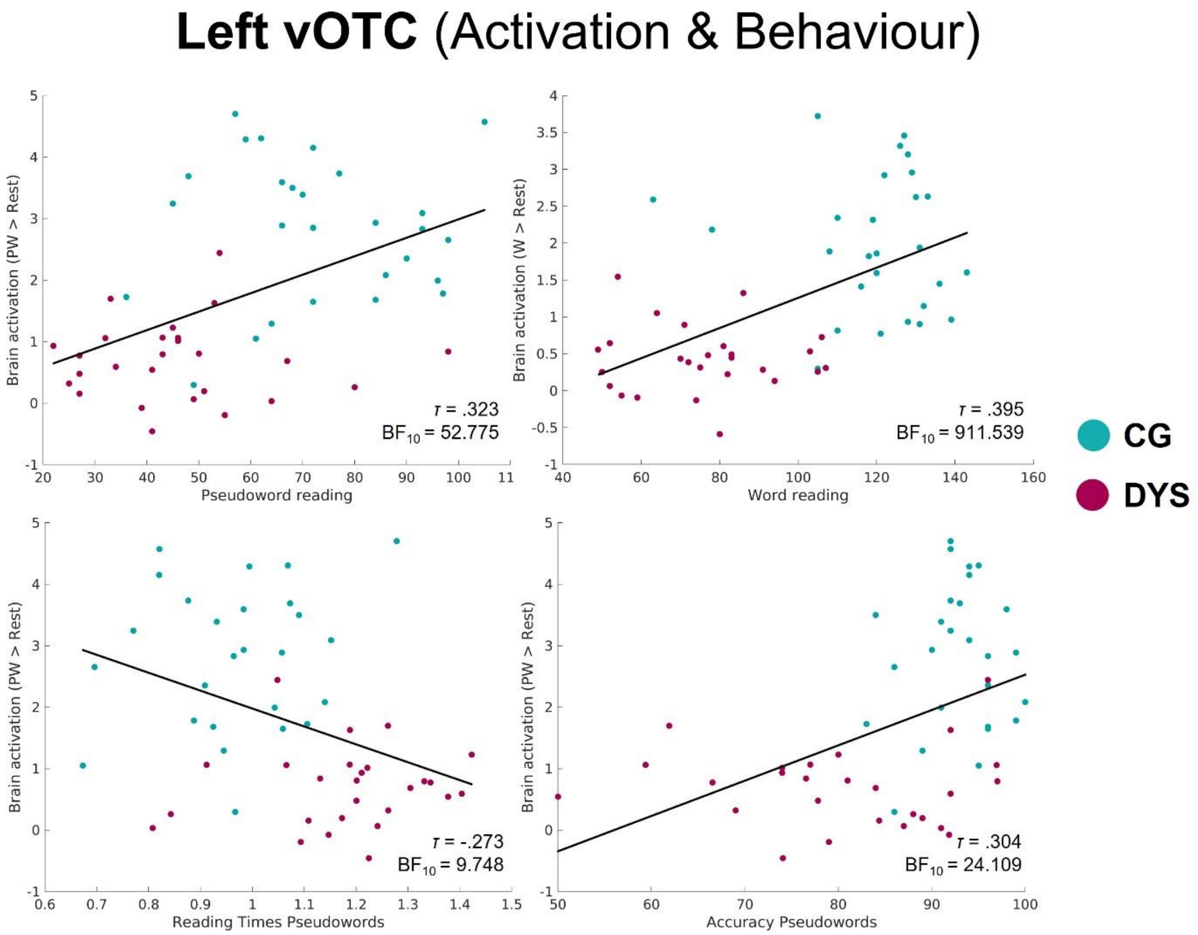

Only robust results as determined through Bayesian correlations (see Table 2 and Supplementary Table 14) are plotted.

Supplementary Figure 6: Correlational plots for functional activation in left SMG for the respective contrasts (pseudowords > rest, words > rest) and behavioural performance (n=53).

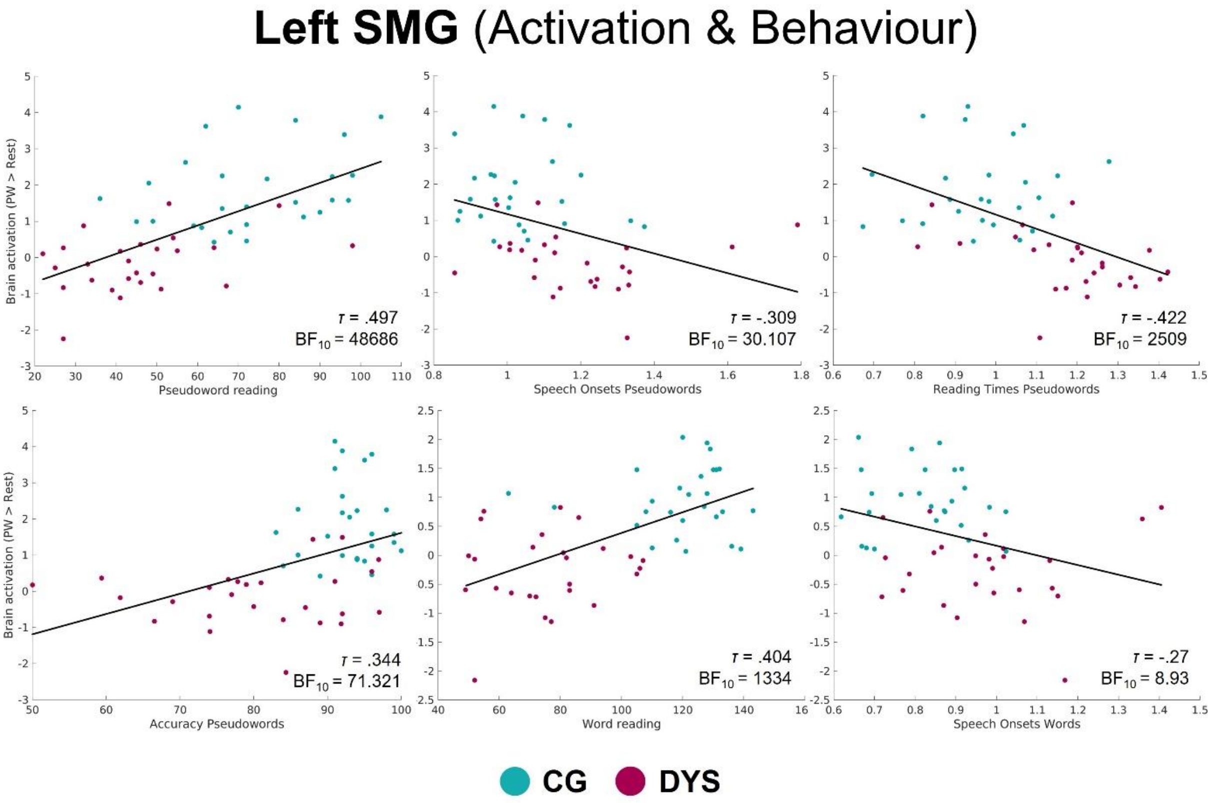

Only robust results as determined through Bayesian correlations (see Table 2 and Supplementary Table 14) are plotted.

Supplementary Figure 7: Correlational plots for functional activation in right vOTC for the respective contrasts (pseudowords > rest, words > rest) and behavioural performance (n=53).

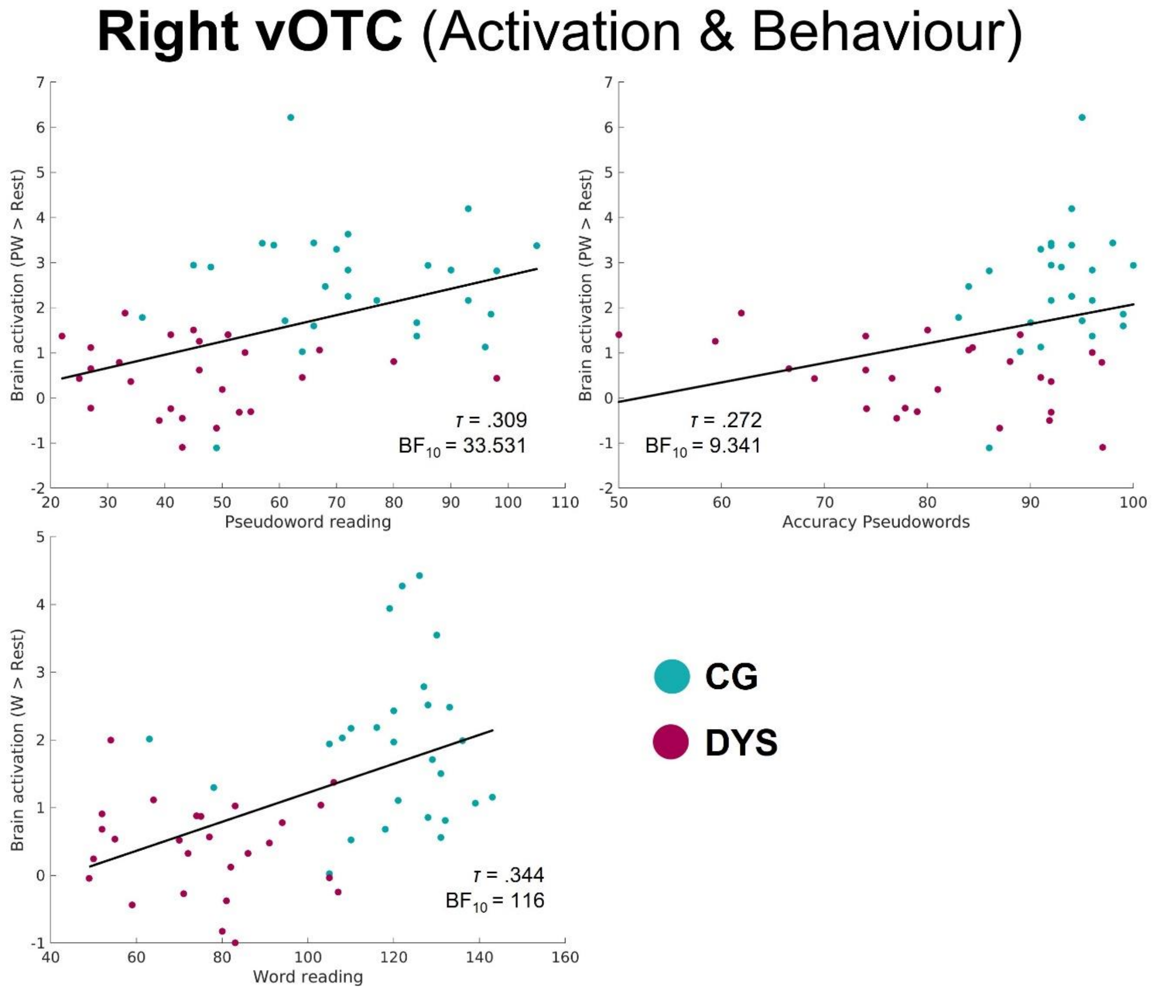

Only robust results as determined through Bayesian correlations (see Table 2 and Supplementary Table 14) are plotted.

Supplementary Figure 8: Correlational plots for functional activation in right cerebellum for the respective contrasts (pseudowords > rest, words > rest) and behavioural performance (n=53).

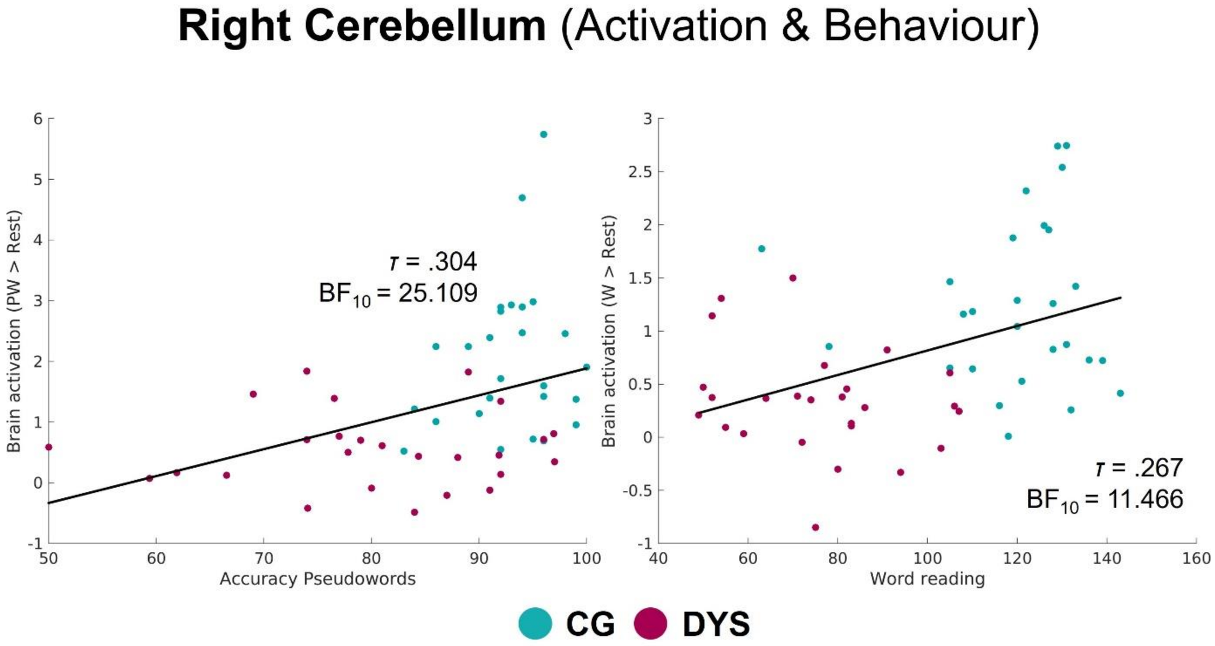

Only one robust result as determined through Bayesian correlations (see Table 2 and Supplementary Table 14) are plotted.

Supplementary Figure 9: Correlational plots for functional activation in the bilateral lingual gyri for the respective contrasts (pseudowords > rest, words > rest) and behavioural performance (n=53).

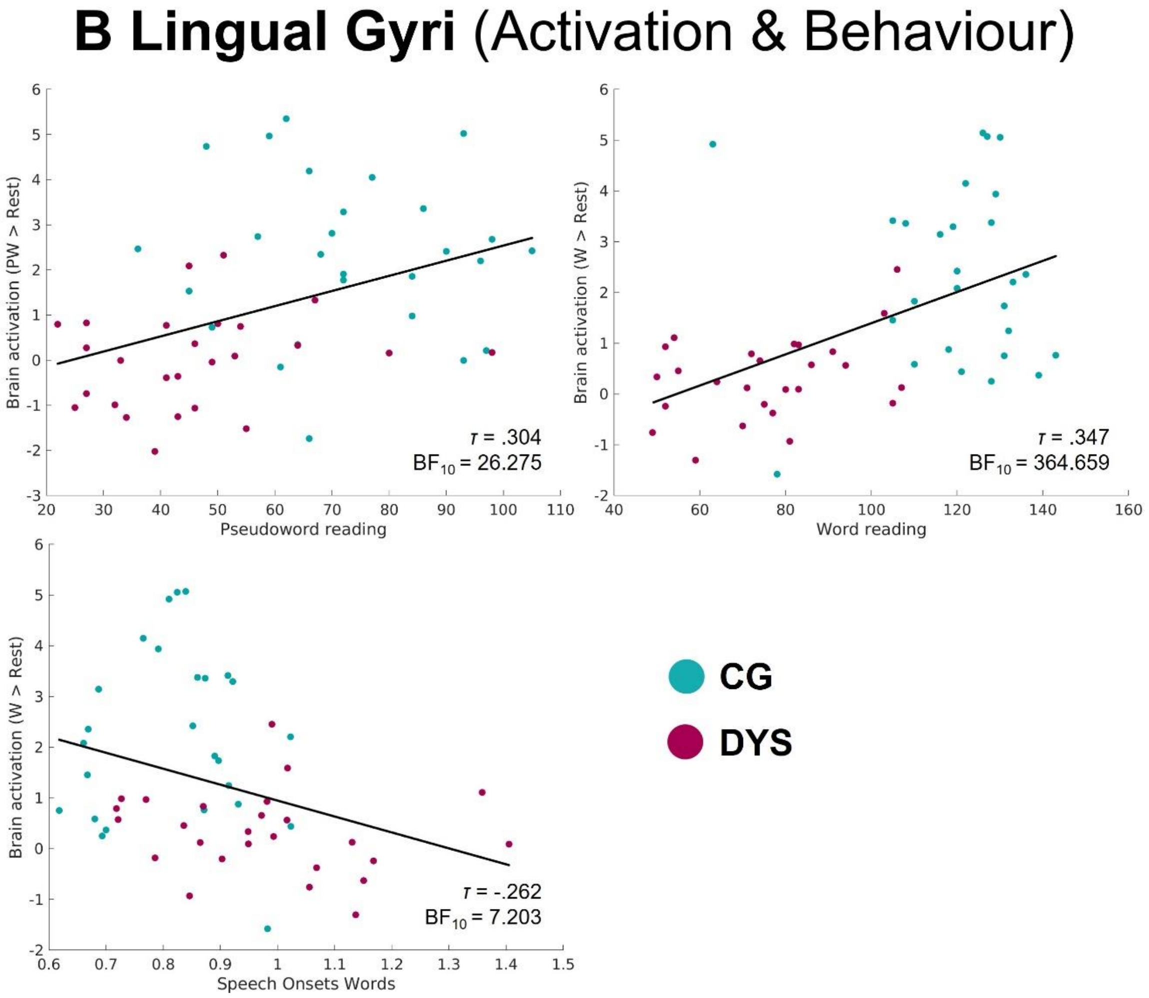

Only robust results as determined through Bayesian correlations (see Table 2 and Supplementary Table 14) are plotted.

Supplementary Figure 10: Robust correlations between individual PPI connectivity based on the respective fMRI contrast (pseudowords > rest, words > rest) and behavioural performance (n=53).

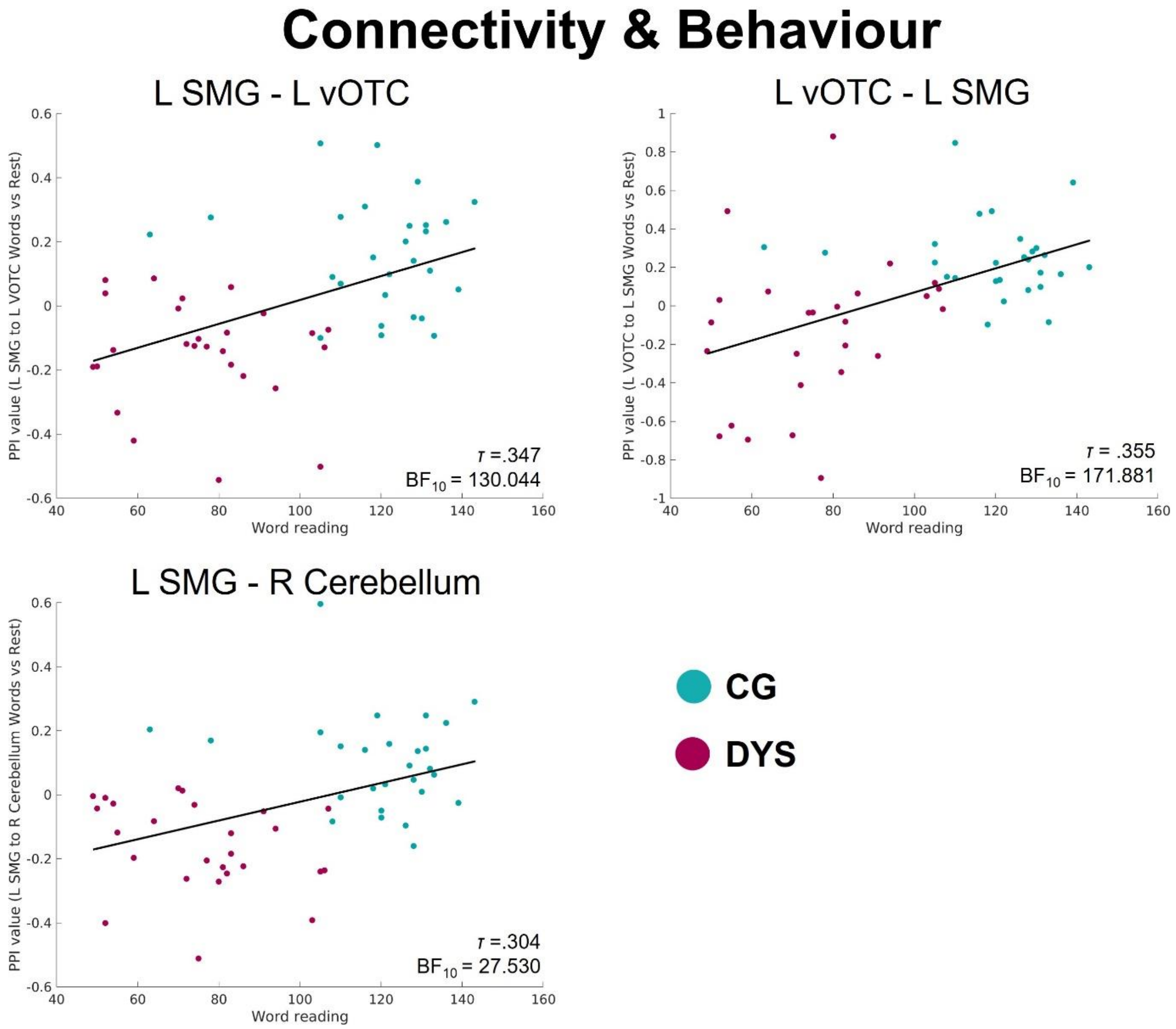

Only robust results as determined through Bayesian correlations (see Table 3 and Supplementary Table 15) are plotted. We find strong links between out-of-scanner word reading performance (as assessed by the SLRT-II) and connectivity between left SMG and left vOTC as well as between left SMG and the right cerebellum during word reading.

Supplementary Figure 11: Robust correlations between connectivity from the right cerebellum seed to other reading-relevant regions during the respective fMRI contrast (pseudowords > rest, words > rest) and behavioural performance (n=53).

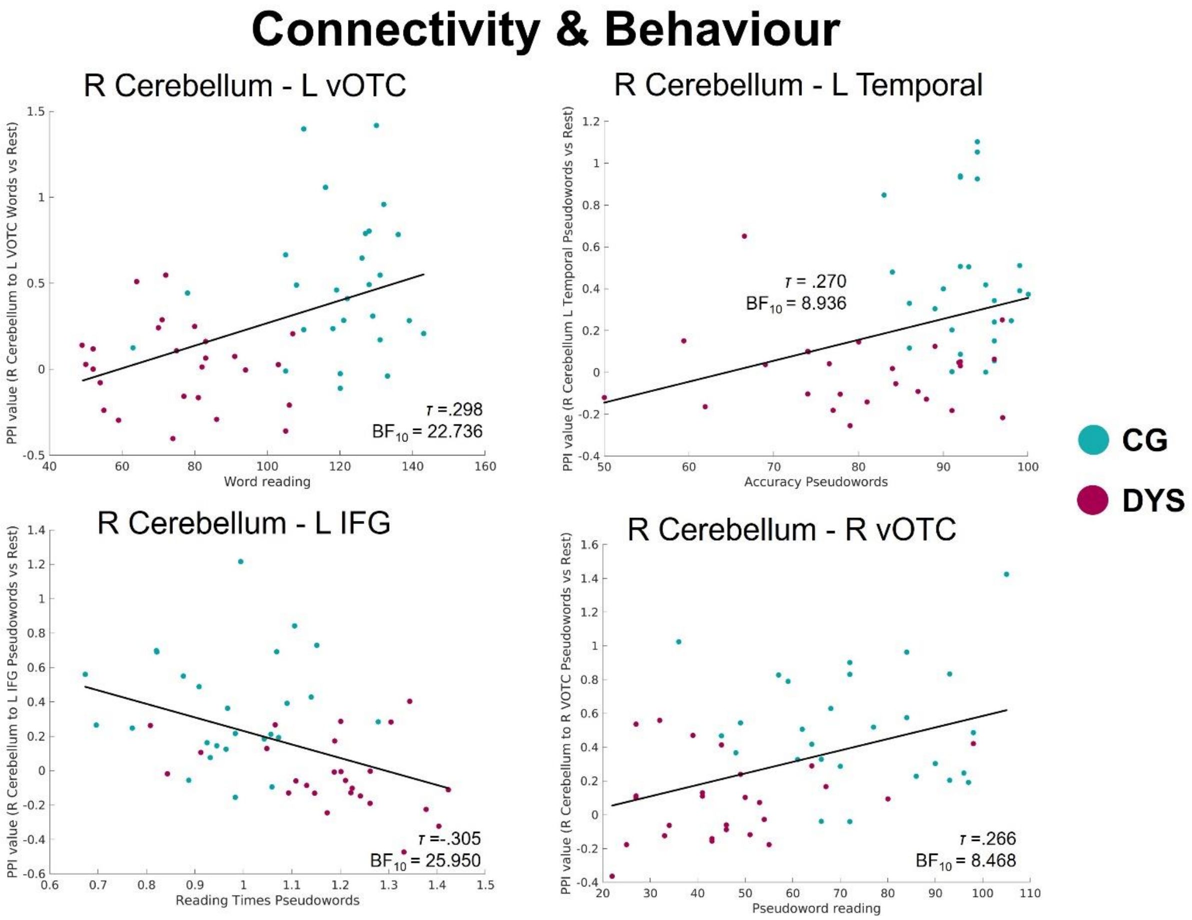

Only robust results as determined through Bayesian correlations (see Table 3 and Supplementary Table 15) are plotted. We find that connectivity from the right cerebellum to the left vOTC is tied to word reading, whereas connectivity to left temporal, inferior frontal and right vOTC regions is tied to pseudoword reading performance in – and outside the scanner.

Supplementary Table 15: Full table of Bayesian correlations for connections of interest derived from PPI analyses and reading performance (n=53). Out-of-scanner performance comprised word and pseudoword reading as assessed by the SLRT-II, whereas in-scanner performance included pseudoword / word speech onsets, reading times and accuracy. Significant results with $BF_{10} > 7$ are provided in Table 3 in the Manuscript.

Connections of interests (PPI)	Performance	τ	BF_{10}
L SMG --> L Cerebellum (words > rest)	word accuracy (MRI)	0.199	1.495
	word reading (SLRT)	0.203	1.704
	word reading times (MRI)	-0.030	0.189
	word speech onsets (MRI)	-0.157	0.673
L SMG --> L VOTC (words > rest)	word accuracy (MRI)	0.212	1.995
	word reading (SLRT)	0.347	130.044
	word reading times (MRI)	-0.012	0.182
	word speech onsets (MRI)	-0.172	0.878
L SMG --> R Cerebellum (words > rest)	word accuracy (MRI)	0.191	1.278
	word reading (SLRT)	0.304	27.53
	word reading times (MRI)	-0.048	0.204
	word speech onsets (MRI)	-0.151	0.609
L vOTC --> L SMG (words > rest)	word accuracy (MRI)	0.124	0.409
	word reading (SLRT)	0.355	171.881
	word reading times (MRI)	0.020	0.184
	word speech onsets (MRI)	-0.176	0.955
L VOTC --> R VOTC (pseudowords > rest)	pseudoword accuracy (MRI)	0.135	0.481
	pseudoword reading (SLRT)	0.210	2
	pseudoword reading times (MRI)	-0.252	5.386
	pseudoword speech onsets (MRI)	-0.208	1.834
R Cerebellum --> L Temp (pseudowords > rest)	pseudoword accuracy (MRI)	0.270	8.936
	pseudoword reading (SLRT)	0.220	2.544
	pseudoword reading times (MRI)	-0.189	1.209
	pseudoword speech onsets (MRI)	-0.124	0.409
R Cerebellum --> L IFG (pseudowords > rest)	pseudoword accuracy (MRI)	0.178	0.987
	pseudoword reading (SLRT)	0.234	3.525
	pseudoword reading times (MRI)	-0.305	25.95
	pseudoword speech onsets (MRI)	-0.104	0.322
R Cerebellum --> L vOTC (words > rest)	word accuracy (MRI)	0.096	0.294
	word reading (SLRT)	0.298	22.736
	word reading times (MRI)	0.072	0.239
	word speech onsets (MRI)	-0.130	0.444
R Cerebellum --> L vOTC (pseudowords > rest)	pseudoword accuracy (MRI)	0.172	0.88
	pseudoword reading (SLRT)	0.266	8.468
	pseudoword reading times (MRI)	-0.237	3.63
	pseudoword speech onsets (MRI)	-0.090	0.279

Supplementary Fig. 12: Schematic summary of correlations between functional activation and functional connectivity with behavioural reading performance

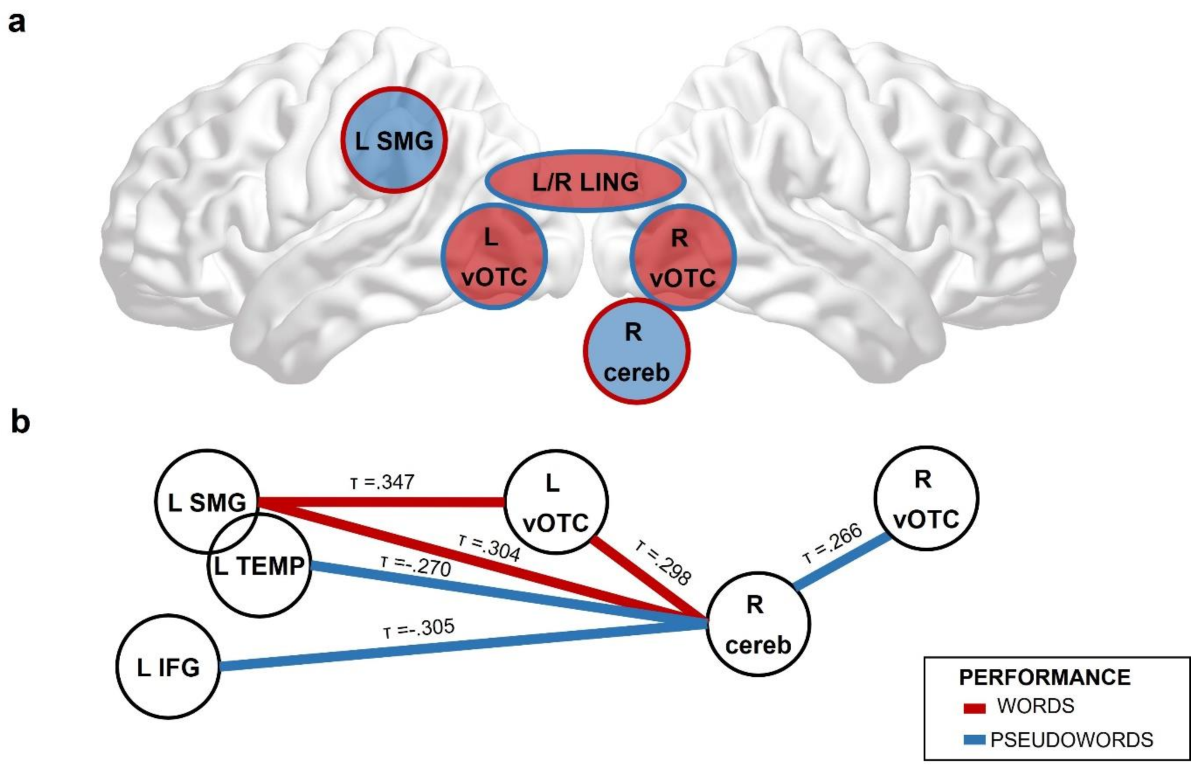

a Areas that show marked hypoactivation for the respective stimuli and two which reading process they are linked to as revealed by correlations with functional activation (red: words; blue: pseudowords). Whereas the left SMG and the right cerebellum were strongly linked to pseudowords, the left vOTC, right vOTC and bilateral lingual gyri (L/R LING) correlated more strongly with words. **b** The triangle between left SMG, left vOTC and right cerebellum showed tight links to word reading performance (red). All connections with the right cerebellum, in contrast, were linked to pseudoword performance (blue). Kendall's τ is reported for significant connectivity-behaviour correlations (n=53).

Supplementary Fig. 13: Schematic illustration of processing along the dorsal reading route in dyslexia based on current DCM findings.

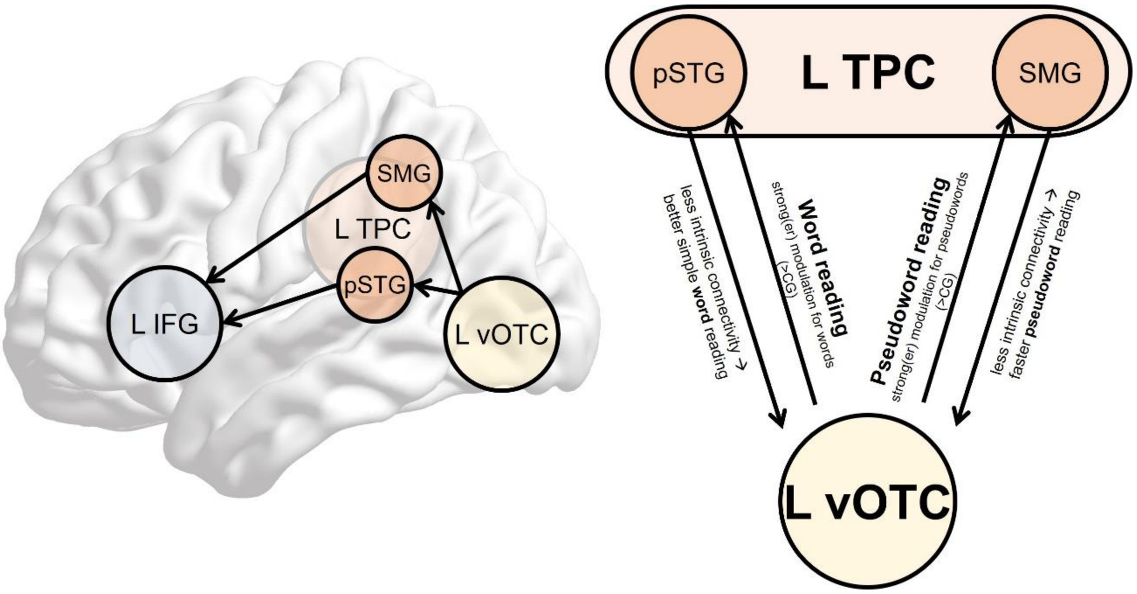

Based on our DCM findings, we suggest that both words and pseudowords are at least partially processed via a dorsal route in dyslexia, but the engagement of TPC subregions might be crucial. Effective connectivity analyses suggest that the left vOTC to left pSTG (TPC) connection was modulated by words, whereas the connection to the left SMG was modulated by pseudowords in dyslexia. Our behavioural correlations support their implication for word and pseudoword reading. Please note that the schematic illustration only displays the specific pathway via the dorsal route and does not imply an altered ventral route or serial processing.

Supplementary Table 16: fMRI motion parameters for all subjects, including the number of motion outliers and maximum (max_FD), mean (mean_FD) and standard deviation (std_FD) of framewise displacement (FD).

Subject	group	num_outliers	max_FD	mean_FD	std_FD
Sub-1	DYS	6	1.2959	0.2581	0.1651
Sub-2	DYS	2	0.9470	0.2917	0.1397
Sub-3	DYS	0	0.6794	0.1942	0.0995
Sub-4	DYS	39	5.9748	0.4150	0.3661
Sub-5	DYS	0	0.8007	0.2588	0.1209
Sub-6	DYS	0	0.7446	0.2026	0.1024
Sub-7	DYS	30	2.0691	0.4069	0.2718
Sub-8	DYS	59	1.7479	0.4661	0.2676
Sub-9	DYS	1	1.0999	0.1714	0.0939
Sub-10	DYS	0	0.6410	0.2076	0.1055
Sub-11	DYS	0	0.4630	0.1667	0.0693
Sub-12	DYS	0	0.4167	0.1490	0.0643
Sub-13	DYS	1	0.9234	0.2681	0.1265
Sub-14	DYS	1	0.9278	0.1920	0.1126
Sub-15	DYS	0	0.4910	0.1866	0.0849
Sub-16	DYS	1	1.0885	0.2065	0.1148
Sub-17	DYS	5	1.3238	0.3810	0.1902
Sub-18	DYS	21	6.1666	0.3328	0.3573
Sub-19	DYS	1	1.1901	0.2220	0.1190
Sub-20	DYS	15	2.9056	0.2339	0.2333
Sub-21	DYS	0	0.4390	0.1884	0.0856
Sub-22	DYS	1	0.9856	0.1970	0.1141
Sub-23	DYS	4	1.3790	0.2834	0.1580
Sub-24	DYS	6	2.6000	0.2080	0.1750
Sub-25	DYS	0	0.8364	0.1641	0.0882
Sub-26	DYS	0	0.8811	0.2253	0.1231
sub-1	CG	0	0.7690	0.2669	0.1273
sub-2	CG	11	1.6991	0.3850	0.1878
sub-3	CG	14	1.4774	0.3425	0.2075
sub-4	CG	2	0.9516	0.2716	0.1602
sub-5	CG	0	0.5382	0.1706	0.0761
sub-6	CG	19	1.5951	0.3422	0.2124
sub-7	CG	0	0.5043	0.2006	0.0832
sub-8	CG	0	0.4668	0.1114	0.0553
sub-9	CG	0	0.8473	0.1644	0.1105
sub-10	CG	0	0.7335	0.2163	0.0978
sub-11	CG	0	0.8504	0.2956	0.1499
sub-12	CG	6	1.8085	0.2695	0.1742
sub-13	CG	0	0.8594	0.2215	0.1325
sub-14	CG	0	0.6814	0.1891	0.0878
sub-15	CG	0	0.8589	0.1680	0.1033
sub-16	CG	0	0.6940	0.1516	0.0862
sub-17	CG	3	1.2210	0.3112	0.1507
sub-18	CG	0	0.8289	0.2679	0.1311
sub-19	CG	0	0.4869	0.1497	0.0793
sub-20	CG	2	2.3739	0.1669	0.1447
sub-21	CG	78	8.3539	0.5476	0.8127
sub-22	CG	0	0.4749	0.1285	0.0703

sub-23	CG	6	1.2003	0.2565	0.1602
sub-24	CG	0	0.5380	0.1234	0.0661
sub-25	CG	1	0.9580	0.2284	0.1209
sub-26	CG	0	0.7583	0.1890	0.1023
sub-27	CG	8	1.4499	0.2650	0.1870
sub-28	CG	0	0.7833	0.2106	0.1129

Supplementary Fig. 14: PPI findings including only 10% of active voxels in the ROI selection process.

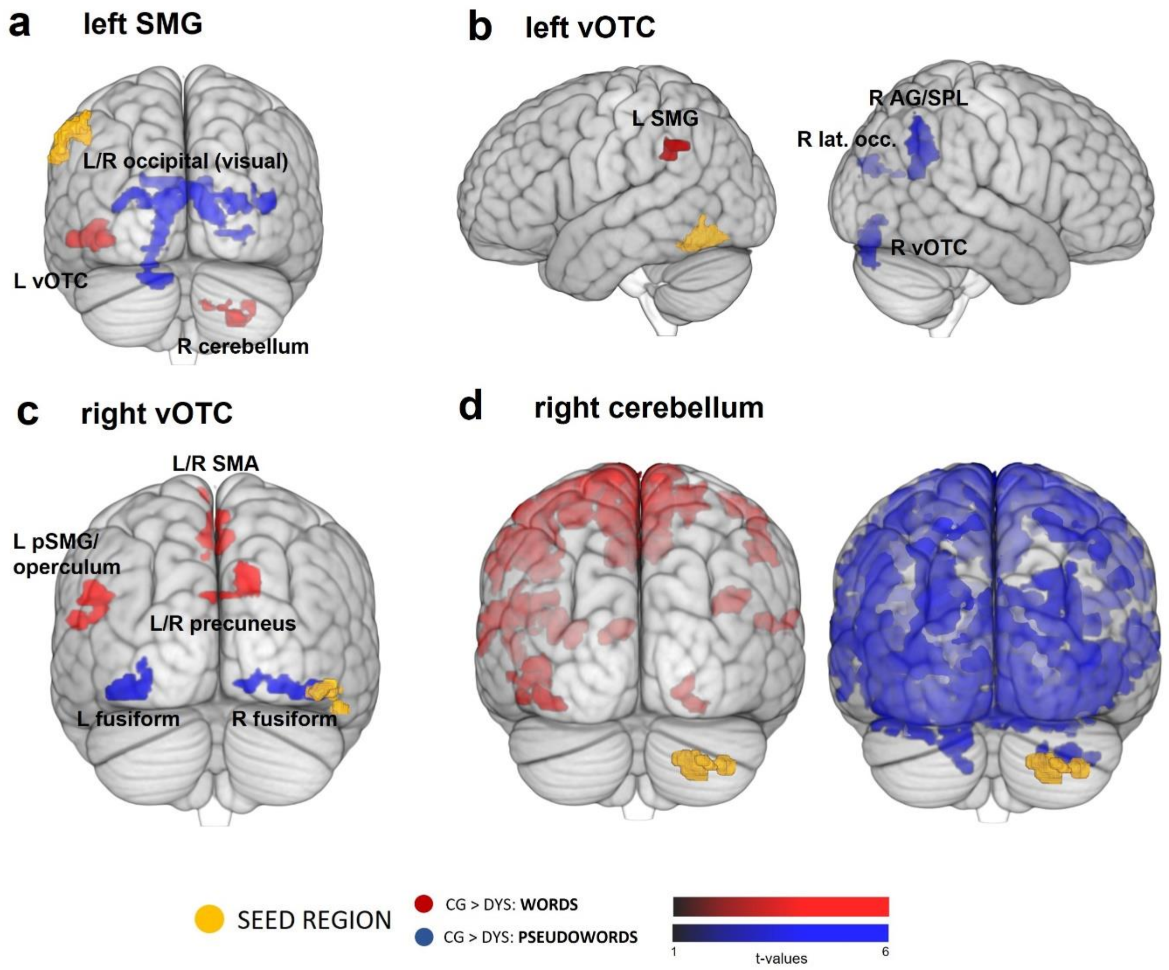

For all panels (**a,b,c,d**), findings resemble the presented findings in the manuscript (Figure 4). For hypoconnectivity from the left vOTC for word processing (**b**), no significant findings were found after cluster correction (FWE p<0.05). At an uncorrected level (p<0.001 voxel-level), three clusters emerged, among which the left SMG cluster was the only one that comprised >15 voxels and is thus shown (**b**).
