# Supplementary material for: Disrupted network interactions serve as a neural marker of dyslexia
Source: Commun Biol. 2023 Nov 3;6:1114. doi: 10.1038/s42003-023-05499-2 (PMC10624919; doi:10.1038/s42003-023-05499-2)
Supplement: Supplementary file 3 — Reporting Summary [file 42003_2023_5499_MOESM3_ESM.pdf]

nature portfolio

Corresponding author(s): Sabrina Turker

Last updated by author(s): Sabrina Turker, 20/10/2023

Reporting Summary

Nature Portfolio wishes to improve the reproducibility of the work that we publish. This form provides structure for consistency and transparency in reporting. For further information on Nature Portfolio policies, see our Editorial Policies and the Editorial Policy Checklist.

Statistics

For all statistical analyses, confirm that the following items are present in the figure legend, table legend, main text, or Methods section.

n/a	Confirmed	
☐	☒	The exact sample size (*n*) for each experimental group/condition, given as a discrete number and unit of measurement
☐	☒	A statement on whether measurements were taken from distinct samples or whether the same sample was measured repeatedly
☐	☒	The statistical test(s) used AND whether they are one- or two-sided<br>*Only common tests should be described solely by name; describe more complex techniques in the Methods section.*
☐	☒	A description of all covariates tested
☐	☒	A description of any assumptions or corrections, such as tests of normality and adjustment for multiple comparisons
☐	☒	A full description of the statistical parameters including central tendency (e.g. means) or other basic estimates (e.g. regression coefficient) AND variation (e.g. standard deviation) or associated estimates of uncertainty (e.g. confidence intervals)
☐	☒	For null hypothesis testing, the test statistic (e.g. *F*, *t*, *r*) with confidence intervals, effect sizes, degrees of freedom and *P* value noted<br>*Give P values as exact values whenever suitable.*
☒	☐	For Bayesian analysis, information on the choice of priors and Markov chain Monte Carlo settings
☐	☒	For hierarchical and complex designs, identification of the appropriate level for tests and full reporting of outcomes
☐	☒	Estimates of effect sizes (e.g. Cohen's *d*, Pearson's *r*), indicating how they were calculated

Our web collection on statistics for biologists contains articles on many of the points above.

Software and code

Policy information about availability of computer code

Data collection	*Provide a description of all commercial, open source and custom code used to collect the data in this study, specifying the version used OR state that no software was used.*
Data analysis	https://osf.io/cy8tk/

For manuscripts utilizing custom algorithms or software that are central to the research but not yet described in published literature, software must be made available to editors and reviewers. We strongly encourage code deposition in a community repository (e.g. GitHub). See the Nature Portfolio guidelines for submitting code & software for further information.

Data

Policy information about availability of data

All manuscripts must include a data availability statement. This statement should provide the following information, where applicable:

- Accession codes, unique identifiers, or web links for publicly available datasets
- A description of any restrictions on data availability
- For clinical datasets or third party data, please ensure that the statement adheres to our policy

Data and code underlying all analyses and figures is provided via the following OSF registry: https://osf.io/cy8tk/ 113 (no password needed).

Research involving human participants, their data, or biological material

Policy information about studies with human participants or human data. See also policy information about sex, gender (identity/presentation), and sexual orientation and race, ethnicity and racism.

Reporting on sex and gender	Number of females and males within the sample are given; did not enter analyses since none of our hypotheses addressed it
Reporting on race, ethnicity, or other socially relevant groupings	no grouping according to such factors took place; i.e., participants were not chosen due to race, ethnicity or social groupings; however, the full sample comprised white, German speaking students or graduates with few exceptions (only concerning education) and 50% of subjects had a prior diagnosis of dyslexia
Population characteristics	age: 18-40y, nonverbal IQ > 85; see below
Recruitment	recruitment strategies database of research institute, flyers, social media
Ethics oversight	The study was approved by the Ethics commitee of the University of Leipzig and follows the guidelines of the Declaration of Helsinki.

Note that full information on the approval of the study protocol must also be provided in the manuscript.

Field-specific reporting

Please select the one below that is the best fit for your research. If you are not sure, read the appropriate sections before making your selection.

☐ Life sciences ☒ Behavioural & social sciences ☐ Ecological, evolutionary & environmental sciences

Behavioural & social sciences study design

All studies must disclose on these points even when the disclosure is negative.

Study description	cross-sectional quantitative analyses
Research sample	N=53; 18-40y German native speakers residing within Germany who were suitable for non-invasive brain stimulation and functional neuroimaging (fMRI); 50% had a prior dyslexia diagnosis
Sampling strategy	sample size was chosen based on previous studies (in the literature and from our own lad) and post-hoc sensitivity analyses were performed
Data collection	behavioural assessment included paper-pencil and computer tests, additionally we performed fMRI (3T scanner)
Timing	October 2020 to March 2022
Data exclusions	One person was excluded due to technical problems with speech recordings in the MRI
Non-participation	Only one participant dropped out after behavioural testing (data was discarded and not taken into account for the whole study)
Randomization	group allocation based on dyslexia status

Dual use research of concern

Policy information about dual use research of concern

Hazards

Could the accidental, deliberate or reckless misuse of agents or technologies generated in the work, or the application of information presented in the manuscript, pose a threat to:

No	Yes	
☒	☐	Public health
☒	☐	National security
☒	☐	Crops and/or livestock
☒	☐	Ecosystems
☒	☐	Any other significant area

Experiments of concern

Does the work involve any of these experiments of concern:

No	Yes	
☒	☐	Demonstrate how to render a vaccine ineffective
☒	☐	Confer resistance to therapeutically useful antibiotics or antiviral agents
☒	☐	Enhance the virulence of a pathogen or render a nonpathogen virulent
☒	☐	Increase transmissibility of a pathogen
☒	☐	Alter the host range of a pathogen
☒	☐	Enable evasion of diagnostic/detection modalities
☒	☐	Enable the weaponization of a biological agent or toxin
☒	☐	Any other potentially harmful combination of experiments and agents

Magnetic resonance imaging

Experimental design

Design type	Task-based event-related mini-block design
Design specifications	2 x 2 x 2 factorial design: stimulus type (pseudowords vs. words), complexity (simple vs. complex), group (control vs. dyslexia). Subjects overtly read 100 words (50 simple, 50 complex) and 100 pseudowords (50 simple, 50 complex). Stimuli were presented in mini-blocks of 5 stimuli.
Behavioral performance measures	In-scanner performance measures: speech onsets, reading times, accuracy for all four conditions (simple words, complex words, simple pseudowords, complex pseudowords). Correltaions with out-of-scanner performance measures: speeded word and pseudoword reading (SLRT-II), text reading speech, accuracy and comprehension (LGVT 5–12+), spelling and phonological processing (spelling test, phoneme substitution task), verbal working memory (digit span forward and backward, nonword span), nonverbal IQ, arithmetic skills and continuous attention test

Acquisition

Imaging type(s)	functional (BOLD-fMRI), structural
Field strength	3 Tesla
Sequence & imaging parameters	gradient-echo EPI; TR=2s, TE=22ms, flip angle: 80°, field of view: 204mm, voxel size: 2.5x2.5x2.5mm, A/P phase encoding
Area of acquisition	whole-brain

Diffusion MRI ☐ Used ☒ Not used

Preprocessing

Preprocessing software	fMRIprep (version 20.2.1)
Normalization	Yes; non-linear transformation to MNI space
Normalization template	MNI152NLin2009cAsym
Noise and artifact removal	Motion regressors (the 6 base motion parameters + 6 temporal derivatives of the motion parameters + 12 quadratic terms of the motion parameters and their temporal derivatives) and the top 10 aCompCor regressors explaining the most variance in physiological noise
Volume censoring	Single-volume regressors for timepoints with a high volume-to-volume movement (framewise displacement > 0.9)

Statistical modeling & inference

Model type and settings: Mass-univariate and multivariate

Effect(s) tested: Differences in functional activation (univariate) and activity patterns (multivariate pattern analysis) between groups during the four conditions (simple words, complex words, simple pseudowords, complex pseudowords). Mass-univariate analyses: (1) Control group: words vs. pseudowords; (2) Control group: simple vs. complex; (3) Dyslexia group: words vs. pseudowords; (4) Dyslexia group: words vs. pseudowords; (5) Control vs. dyslexia group for all trials > rest; (6) Control vs. dyslexia group for pseudowords > words. Multivariate pattern analysis: Control vs. dyslexia group for all trials.

Specify type of analysis: ☐ Whole brain ☐ ROI-based ☒ Both

Anatomical location(s): The hypoactive areas from the univariate analyses were taken as ROIs and we compared activation for all four conditions (simple words, complex words, simple pseudowords and complex pseudowords) in the two groups

Statistic type for inference (See Eklund et al. 2016): Cluster-wise correction

Correction: voxel-wise $p < 0.001$ and cluster-wise $p < 0.05$ FWE-corrected

Models & analysis

n/a	Involved in the study
☐	☒ Functional and/or effective connectivity
☒	☐ Graph analysis
☐	☒ Multivariate modeling or predictive analysis

Functional and/or effective connectivity: Generalized psychophysiological interaction (PPI) analyses: Seeds = left vOTC, right vOTC, left SMG, right cerebellum. Contrasts: (1) Control vs. dyslexia group for words; (2) Control vs. dyslexia group for pseudowords.
Dynamic causal modeling (DCM): Model 1 (classical reading network): left IFG, left TPC, left vOTC. Model 2 (extended reading network): left SMG, left vOTC, right vOTC, right cerebellum. Full model = all regions bidirectionally connected, all trials could drive all regions, all between-region connections could be modulated by both words and pseudowords. Full models were reduced to most likely models given fMRI data. Bayesian model average was computed by taking the average over models weighted by the model evidence. For each model, we estimated a DCM within each group (control group, dyslexia group), as well as the contrast between groups.

Graph analysis: *Report the dependent variable and connectivity measure, specifying weighted graph or binarized graph, subject- or group-level, and the global and/or node summaries used (e.g. clustering coefficient, efficiency, etc.).*

Multivariate modeling and predictive analysis: Multivariate pattern analysis: Control vs. dyslexia group (across all trials). Searchlight decoding analysis using The Decoding Toolbox (https://sites.google.com/site/tdtdecodingtoolbox/home) implemented in Matlab (version 2021a). Searchlight: 5-mm radius sphere. Classifier: L2-norm support vector machine (C=1). Leave-two-participants-out cross validation (one participant per group), yielding 26 cross-validation folds. For statistical inference, we performed a permutation test across the accuracy-minus-chance maps of the different CV-folds (using SnPM13; http://www.nisox.org/Software/SnPM13/), thresholded at a voxel-wise $p < 0.001$ and a cluster-wise $p < 0.05$ FWE-corrected.
